# Dialysis vintage time has the strongest correlation to psychosocial pattern of oral health-related quality of life – a multicentre cross-sectional study

**DOI:** 10.4317/medoral.22624

**Published:** 2018-11-21

**Authors:** Gerhard Schmalz, Marit Dietl, Radovan Vasko, Gerhard A. Müller, Lars Rothermund, Frieder Keller, Dirk Ziebolz, Franz-Maximilian Rasche

**Affiliations:** 1 Department of Cariology, Endodontology and Periodontology, University of Leipzig, Germany; 2 Die Zahnärzte Steinpleis MVZ GmbH, Werdau, Germany; 3 Department of Nephrology and Rheumatology, University Medical Center, Goettingen, Germany; 4 KfH Kuratorium for Dialysis and Kidney Transplantation, Ulm, Germany; 5 Department of Internal Medicine I, Division of Nephrology, University Hospital of Ulm, Ulm, Germany; 6 Department of Internal Medicine, Neurology, Dermatology, Clinic for Endocrinology, Nephrology, Section of Nephrology, University Leipzig, Leipzig, Germany; 7 KfH, Dialysis care unit Kulmbach, Germany

## Abstract

**Background:**

Aim of this cross-sectional, multicentre study was to investigate associations of dialysis vintage time in haemodialysis (CKD5D) patients with oral health-related quality of life (OHRQoL) and dental and periodontal treatment need.

**Material and Methods:**

CKD5D patients were divided into subgroups according to dialysis vintage time in different dialysis centres in Germany. OHRQoL was assessed with oral health impact profile (OHIP-G14). Dental treatment need was classified as presence of carious lesions. Periodontal treatment need was defined as periodontal screening index score (PSI) 3-4.

**Results:**

In total, 190 participants were divided into the subgroups according to the time on CKD5D: 0 - 2 (n = 29), 3 - 5 (n = 35), 6 - 8 (n = 34), 9 - 12 (n = 29), 13 - 20 (n = 34) and >20 years (n = 29). The overall treatment need in the total cohort was 92% (dental 56%, periodontal 88%) with a total OHIP-G14 sum score of 4.17 [2; 0-5] without a significant correlation. Time on CKD5D was inversely correlated with the OHIP G14 score (*p*<0.01, R = -0.201). The pattern psychosocial impact was significantly associated with the dialysis duration (*p*<0.01) and showed a negative correlation to the OHIP-G14 (R = -0.283, Spearman´s rho test *p*<0.01). For oral function also a negative correlation with OHIP-G14 was detected (Spearman´s rho: -0.183).

**Conclusions:**

Patients with a prolonged dialysis vintage time show an improved OHRQoL, which might be mainly caused by the positive development of psychosocial pattern of OHRQoL. The oral health situation of HD patients seems unsatisfying, independently of dialysis vintage time and OHRQoL. Accordingly, an improvement in oral health situation of CKD5D patients is mandatory necessary. Thereby, consideration of psychosocial aspects especially at the beginning of CKD5D therapy and a sensitization regarding oral health issues with increasing vintage time might be recommendable.

** Key words:**Dental care, oral health, oral related quality of life, haemodialysis, chronic kidney disease.

## Introduction

Patients with chronic kidney disease and haemodialysis (CKD5D) must be seen as at high risk patients in dental practice due to systemic cause of renal disease, their general deficiencies and a compromised immune system (1,2). Additionally, deficiencies in oral health, oral hygiene and oral health behaviour of HD patients were described in literature (1-5). Therefore, an improvement in dental care appears necessary. However, CKD5D patients are a very complex patient group with a high physical and also psychological burden caused by their underlying kidney disease (6).

In this context, the quality of life plays an increasing role in CKD5D patients, as it is a main goal of the successful therapy (7). Different studies report a reduced quality of life in this patients group (8). This quality of life is additionally influenced by the duration of dialysis therapy, whereby a deterioration of the quality of life with increasing time on HD has been reported (9).

The oral health-related quality of life (OHRQoL), which is a part of the general quality of life (10), has also been proven in CKD5D patients. The literature showed contradictory results regarding this issue (11-14). The majority of studies, including a previous study by this working group, found normal OHRQoL, irrespective of the high prevalence of dental and periodontal diseases (11-13). However, the influence of the time on CKD5D on OHRQoL is still unclear. Only one study is available, which investigated general quality of life and oral status depending on dialysis duration and found poor oral health and reduced quality of life in CKD5D patients with a prolonged dialysis period (15).

Regularly, oral diseases affect OHRQoL (16,17). Furthermore, oral health was reported to get worse with prolonged CKD5D therapy (18). Accordingly, the OHRQoL depending on dialysis duration appears to be a question of interest. Especially the different patterns including oral function and psychosocial impact (19) might help to understand the complexity and to uncover main influential factors in CKD5D patients. Therefore, aim of this cross-sectional multicentre study was to investigate OHRQoL depending on dental and periodontal treatment need as well as dialysis duration in CKD5D patients. Thereby, the differentiated investigation of OHRQoL considering the oral function and the psychosocial burden should be performed to get information whether oral diseases, psychological burden or a combination would be most relevant in this patient group. Taking the literature into account, it was hypothesized that high treatment need and reduced OHRQoL would be associated with prolonged dialysis duration. Thereby both, oral function and psychosocial burden should be of relevance.

## Material and Methods

The patients for this multicentre study were included from different patient groups of three dialysis centres in Baden-Wuerttemberg (KfH Kuratorium für Dialyse und Nierentransplantation e.V, Ulm and KfH Kuratorium für Dialyse und Nierentransplantation e.V, Ehingen) and Lower Saxony (Department of Nephrology and Rheumatology, University Medical Center Goettingen, Kidney-Rheuma-Center Goettingen, Medical Center Bad Bevensen, Nephrology Medical Center Uelzen, Dialysis and Diabetes Practice Lüneburg), Germany. Only patients with the highest stage of chronic kidney diseases that mandatory requires dialysis (CKD5D) who had exclusively undergone haemodialysis were recruited.

The following information was obtained from the medical record of the study participants: age, gender, diabetes status, smoking habits (smoker or non-smoker for at least five years). Furthermore, if applicable, the date when dialysis therapy started was recorded.

Moreover, patients were divided into subgroups according to their time on haemodialysis. Subgroups were composed based on equal percentiles into six subgroups: 0-2 years, 3-5 years, 6-8 years, 9-12 years, 13-20 years, and >20 years. No matching was performed during group composition. A minimum size of 25 patients each group was aspired.

Mandatory conditions for the participation were the regular haemodialysis therapy in one of the above mentioned dialysis centres, a minimum age of 18 years and voluntary participation in the study. The following exclusion criteria were formulated: impossible oral examination because of poor overall health, drug addicts, cerebral seizure disorders, infectious diseases (hepatitis A, B, C; tuberculosis and HIV) and pregnancy. As further specific criteria, the German language abilities to answer the questionnaire and the presence of remaining teeth were considered.

This cross-sectional study was reviewed and approved by the Ethics Committee of the University Medical Center in Goettingen (No. 43/9/07) and of the central ethic committee of the KfH, Neu-Isenburg. All investigated study participants were informed verbally and in writing about the study and gave their written informed consent. The guidelines for ethical approvals for human subjects were followed in accordance with the Declaration of Helsinki.

-Oral examination 

The oral examination was performed in the Department of Preventive Dentistry, Periodontology and Cariology of the University Medical Center Goettingen or in the participating dialysis facilities. Thereby, a dental and periodontal examination was performed. During dental examination, the number of decayed, missing and filled teeth was assessed using a mirror and probe. Teeth with a reasonable cavitation in the dentine layer were assigned to the D (= decayed) component; filled or crowned teeth were characterised as component F (=filled) and missing teeth were assigned to the M (=missing) component. The DMF-T enables conclusions regarding the caries experience of an investigated individual. Furthermore, the degree of caries restoration (%) was calculated: ratio of filled teeth (FT) to the carious (DT) plus filled teeth (FT) (FT/ (DT+FT) x 100) (20). The periodontal examination was performed using a periodontal probe (PCP 15; Hu-Friedy, Chicago, IL, USA) for measurement of periodontal probing depth (PPD).

Based on these findings, the treatment need was calculated. The presence of carious leasons (D-T) was the marker for the presence of dental treatment need. According to PSR/PSI (21), a PPD of ≥3.5mm was the predictor of periodontal treatment need. The overall treatment need was defined as the presence of dental and/or periodontal treatment need.

-Oral Health Impact Profile (OHIP G14)

The German short form of the Oral Health Impact Profile (OHIP G14) was used to assess the oral health-related quality of life (22,23). The OHIP G14 is a standardized and validated questionnaire, which indicates the frequency of 14 functional and psychosocial impacts that individuals have experienced in the previous month as a result of problems with their teeth, mouth or dentures. Different graduated answers were possible: 0 = “never”, 1 = “hardly ever”, 2 = “occasionally”, 3 = “fairly often” and 4 = “very often”. According to John *et al.* 2016, the different questions were categorized into the patterns “oral function” and “psychosocial impact” (19,24). In accordance to Reissmann et al, 2008, differences in OHIP-G14 values of on average 2 or more points were seen as clinical relevant (25).

-Statistical analysis 

For statistical analysis, SPSS statistical package version 24.0 (SPSS Inc., Chicago, Illinois, US) was used. For the comparison of two non-normal distributed, independent samples, the Mann-Whitney-U-test was applied. For comparison of two non-normal distributed variables with more than two steps the Kruskal-Wallis test was used. Categorical samples were analysed with chi-square test. A bivariate correlation of two metric variables was performed with Spearman´s rho test. Furthermore, a Bonferroni correction for multiple testing was applied. If not indicated otherwise, data are given as median values with range (minimum to maximum), or mean and with standard deviation (± SD). The significance level was determined as *p*<0.05.

## Results

-Patients

A total of 210 patients with a mean age of 64.92 ± 15.7 years were included in the study. Of these patients, the dialysis duration was known for 190 participants, which were divided into the subgroups: 0-2 years (n=29), 3-5 years (n=35), 6-8 years (n=34), 9-12 years (n=29), 13-20 years (n=34) and >20 years (n=29). Between the subgroups, age, gender, smoking habits were comparable, while only diabetes status was different between subgroups (*p*=0.01,  Table 1).

**Table 1:**
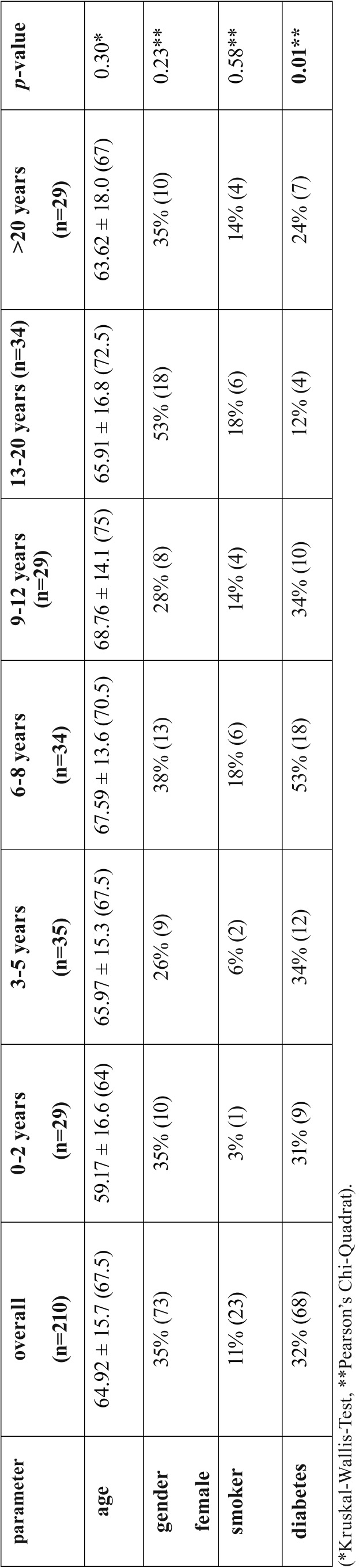
Patient characteristics, smoking habits, diabetes status and dialysis vintage time. Values are given as mean value ± standard deviation (median) or as % (n). Significance level: *p*<0.05. (*Kruskal-Wallis-Test, **Pearson’s Chi-Quadrat).

-Oral examination

The overall treatment need in the total cohort was 92% (dental 56%, periodontal 88%). Patients with a shorter dialysis duration (0-2 years, 3-5 years, 6-8 years and 9-12 years) showed a higher periodontal treatment need with a range between 88%-100% compared to patients with a long dialysis duration (13-20 years and >20 years, 79%, *p*=0.04;  Table 2). The further dental parameters including number of remaining teeth, DMF-T, PPD, degree of caries restauration as well as dental and overall treatment need showed no significant associations to dialysis duration ( Table 2).

**Table 2:**
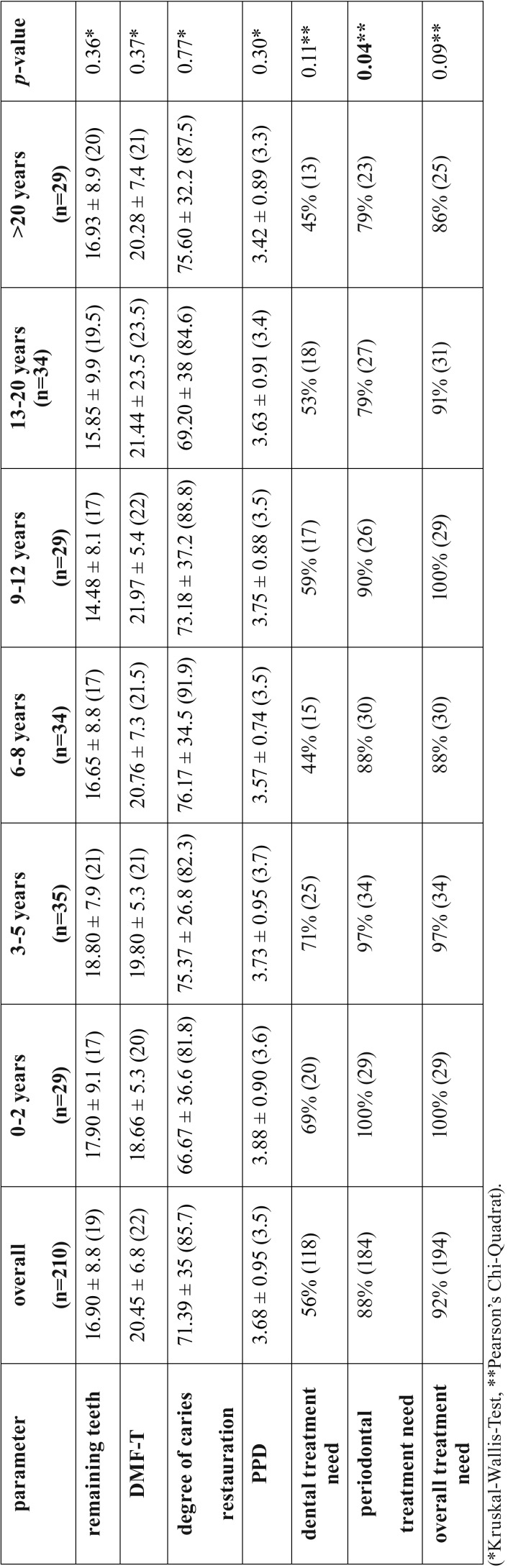
Dental and periodontal findings, treatment need in total cohort and dialysis vintage time. Values are given as mean value ± standard deviation (median) or as % (n). Significance level: *p*<0.05. (*Kruskal-Wallis-Test, ** Pearson’s Chi-Quadrat).

-OHIP G14

**Figure 1:**
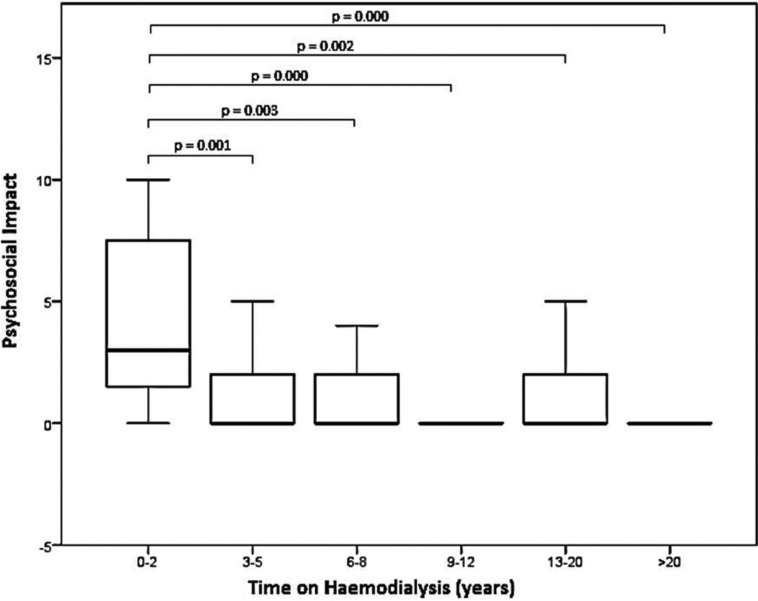
Differences in OHIP G14 pattern “psychosocial impact” between the subgroups. Statistical significant results after Bonferroni correction are highlighted.

**Figure 2:**
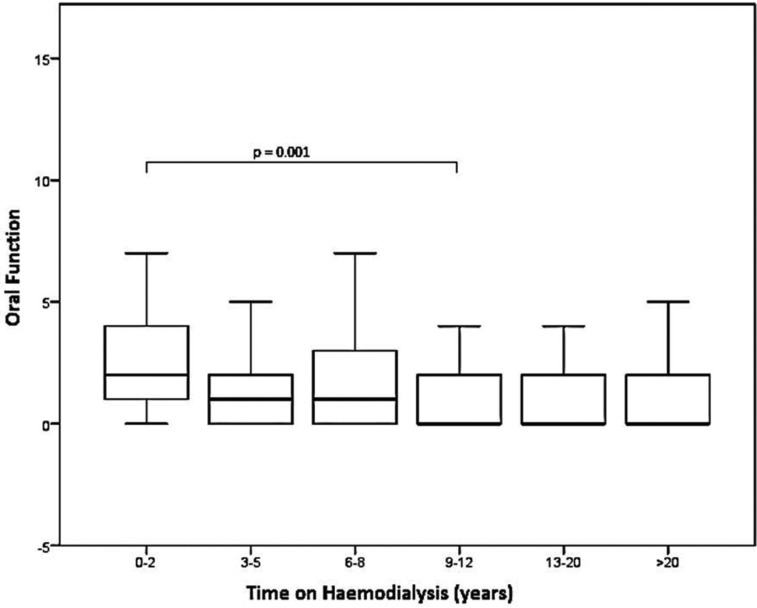
Differences in OHIP G14 pattern “oral function” between the subgroups. Statistical significant results after Bonferroni correction are highlighted.

The findings of OHIP-G14 are shown in  Table 3. The mean OHIP-G14 sum score of the total cohort was 4.17 [2; 0-5]. Patients with a short dialysis duration showed statistically significant and clinically relevant higher OHIP G14 scores than patients with a long dialysis duration (*p*<0.01). Regarding the singular patterns, all questions of psychosocial impact beside of “feeling of tension” (*p*=0.51) were significantly lower in patients with long compared to short dialysis duration (*p*<0.05). The overall pattern psychosocial impact was significantly associated with the dialysis duration (*p*<0.01, Fig. 1) and showed a negative correlation (Spearman´s rho: -0.283). For oral function, only “interrupting meals”, “uncomfortable to eat” and the overall pattern oral function showed significant association to dialysis duration (*p*<0.05, Fig. 2) and also a negative correlation was detected for oral function (Spearman´s rho: -0.183).

**Table 3:**
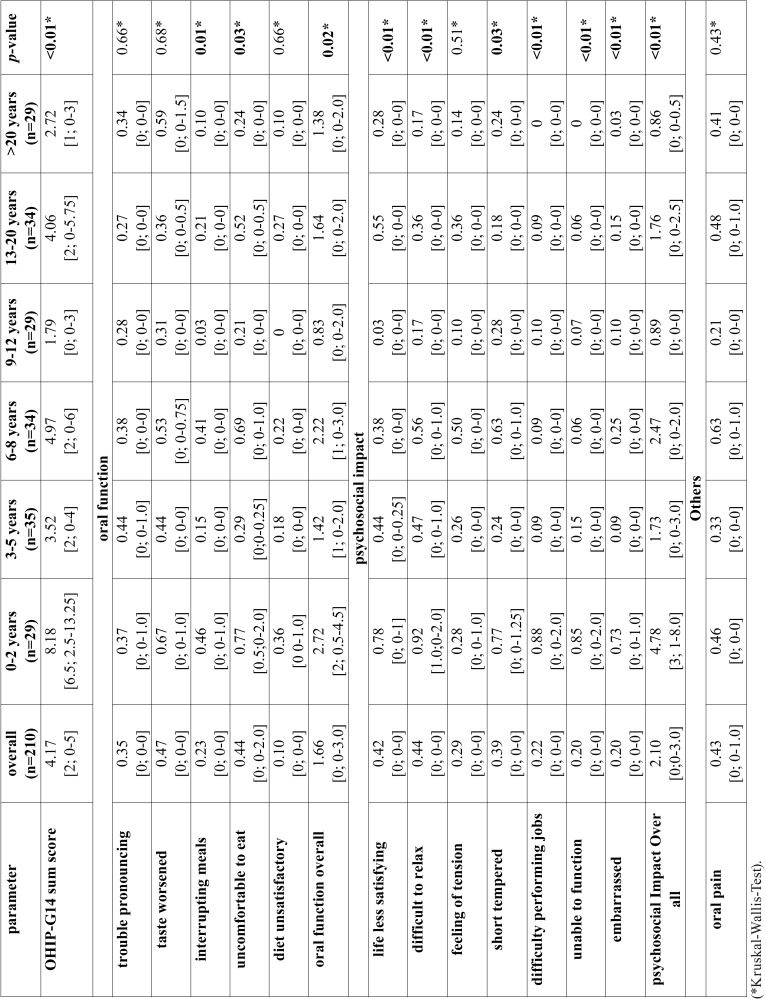
Analysis of the different patterns of OHIP-G14 results in total cohort and between subgroups according to time haemodialysis (mean [median, 25-75 percentile]). Significance level: *p*<0.05 (*Kruskal-Wallis-Test).

Of the potential investigated influential factors on the OHIP G14 scores, the dialysis duration was clinically relevant and statistically significantly associated (*p*<0.01) and negatively correlated to the OHIP G14 score (Spearman´s rho: -0.201). Furthermore, the PPD was clinically relevant and statistically relevant associated (*p*=0.03) and correlated with OHIP G14 scores (Spearman´s rho: 0.164). No further associations to dental or general parameters with the OHIP G14 scores were detected ( Table 4).

**Table 4:**
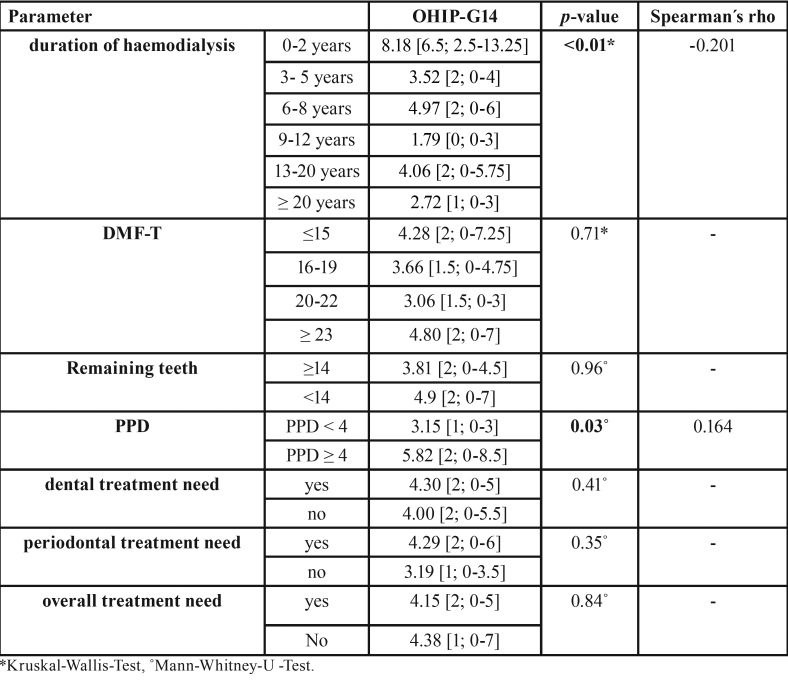
Analysis of different potential influence factors on OHIP-G14 values of patients haemodialysis (mean [median, 25-75 percentile]). Significant results are highlighted in bold. Significance level: *p*<0.05.(*Kruskal-Wallis-Test, ˚Mann-Whitney-U –Test).

## Discussion

Of all investigated potential influential factors on OHIP G14 scores, the overall time with CKD5D was significantly inversely correlated with OHIP G14 score. Thereby, the pattern psychosocial impact showed a stronger correlation to the time on HD than the pattern oral function.

The oral health situation of HD patients has repeatedly been reported to be insufficient (1-5). Therefore the current study reported and investigated the treatment need of included patients, whereby an overall treatment need of 92% (dental 56%, periodontal 88%) was detected. Although CKD5D patients are categorized as at-risk patients due to their general disease burden and resulting immunological impairments (1,2), the detected treatment need is even higher than in general population in Germany (26). Interestingly, this high treatment need showed no association to OHRQoL of these patients. This is in accordance to previous studies by this working group, showing that the OHRQoL is independent from of oral condition for different at risk patient groups (13,27-29). The OHRQoL was the main focus of the current study. In the total cohort, a total OHIP-G14 score of 4.17 was detected. This is a little higher compared to CKD5D patients in the previous study (13). Three further studies, which investigated OHRQoL of HD patients are available, which showed inconsistent results. In accordance to the current study, Hajian-Tilaki *et al.* demonstrated good OHRQoL, although poor oral health was found (11). Similarly, Guzeldemir *et al.* found comparable results in a Turkish CKD5D group (12). Solely Pakpour *et al.* demonstrated poor dental health in combination with reduced OHRQoL in HD patients (14). Taking reference values for healthy population into account, which have been defined by John *et al.* 2004, the OHIP-G14 score in the current study lies just negligible over the mentioned range between 0 and 4 for healthy fully or partially dentate individuals (23).

One novel approach was the investigation of time on CKD5D on OHRQoL in total and on two major patterns, the oral function and the psychosocial impact. It has been reported, that oral health (18) and general quality of life (9) is getting worse the longer a patient stays under CKD5D therapy. Accordingly, a recent investigation by Andrade *et al.* 2017 concluded that poor oral health might reflect reduced quality of life of CKD5D patients receiving CKD5D for a prolonged time period (15). In contrast to these findings of the literature, the current study showed OHRQoL to be better in patients with prolonged CKD5D therapy.

Taken together, the OHIP-G14 total score and patterns of the oral function and the psychosocial impact between the different subgroups allow different hypotheses. On the one hand, it is conceivable, that especially patients short term under CKD5D might feel an enormous psychological burden, resulting in impaired psychosocial pattern of OHRQoL. With longer time under CKD5D, patients might get more familiar with the situation, resulting in lower impairment of psychosocial impact. However, this would be in contrast to the literature, showing quality of life getting worse during CKD5D therapy (9). Thereby it has to be considered, that most studies investigated comparably short time periods, while the current study maps a very long time span of more than 20 years. On the other hand, the overall reduction of general quality of life might affect the OHRQoL in the way that patients do not attach importance to their oral condition any more due to their growing general burden, resulting in reduced impact on OHRQoL. This is supported by the reduced affection of pattern oral function without improvement of oral health situation. In contrast, the fact that OHRQoL is part of the overall quality of life (10) is conflicting with this hypothesis.

This is the first study investigating OHRQoL and the singular patterns oral function and psychosocial impact depending on oral health parameters and dialysis duration in CKD5D patients. The inclusion of 210 patients and more than 25 participants each subgroup with a comparable age, gender and smoking habit is a further strength of the investigation. Nevertheless, the study has several limitations. The design as a cross-sectional study limits the possible conclusion of the examination. To detect the influence of dialysis duration on the OHRQoL, a longitudinal design would be necessary. Accordingly, it must be stated that all the presented results are purely correlation and not cause-effect results. Furthermore, a previously performed power calculation would have been helpful, but was omitted and it was tried to include as many available patients as possible. Patients under CKD5D are hard to recruit and a difficult, complex patient group with a high general burden. Furthermore, assessment of oral hygiene parameters and dental behaviour as well as general quality of life could have strengthened the results and would have been helpful to prove the hypotheses formed in the discussion. Moreover, the question whether the level of dehydration and potentially the presence of xerostomia would be involved in oral health and OHRQoL would be of interest and should be considered for further studies in this field. Similarly, the potential influence of causal underlying diseases on OHRQoL could be an interesting aspect for future investigations. Additionally, the difference in diabetes status between subgroups is a limitation, but diabetes status was already shown to have no influence on OHRQoL (30). The differences in Health Systems of different countries may be an independent factor that should be considered in the interpretation of the results, too.

Nevertheless, the current study´s results demonstrate once more the necessity of improvement in oral care of CKD5D patients and the relevance of psychosocial factors regarding this issue, making an interdisciplinary collaboration a mandatory perquisite.

## Conclusions

Within the limitations of the study, the dialysis vintage time showed the strongest correlation to psychosocial pattern of OHRQoL, whereby especially patients short-term under CKD5D had worse OHRQoL. Furthermore, OHRQoL showed no associations to their high dental and periodontal treatment need.
